# The use of romosozumab versus denosumab in patients with osteoporosis

**DOI:** 10.1590/1806-9282.2025D719

**Published:** 2025-10-17

**Authors:** Idevaldo Floriano, Antonio Silvinato, Wanderley Marques Bernardo

**Affiliations:** 1 Brazilian Medical Association, Evidence-Based Medicine – São Paulo (SP), Brazil.; 2 Universidade de São Paulo, Faculty of Medicine – São Paulo (SP), Brazil.

## INTRODUCTION

Osteoporosis is one of the main causes of morbidity and mortality among the elderly and individuals at high risk, predominantly affecting women, although it also affects men to a lesser extent. The elderly population is the age group most affected by the disease, which represents one of the main causes of hospitalization. It is estimated that up to 30% of patients with femoral fractures will die in the first year after the event^1^.

It is an osteometabolic disease characterized by the progressive reduction of bone mass, accompanied by changes in the microarchitecture of bone tissue, which results in greater fragility and increased risk of fractures. The main risk factors include: inadequate diet (low calcium and vitamin D intake), sedentary lifestyle, excessive alcohol consumption (which interferes with calcium absorption and parathyroid hormone metabolism), smoking, use of certain medications (such as corticosteroids, anticonvulsants, and chemotherapy), hormonal diseases (hyperparathyroidism, hypogonadism in men, and hypoestrogenism), and malnutrition.

The diagnosis is made by means of bone mineral density (BMD), using the technique of dual-energy X-ray absorptiometry (DEXA), considered the gold standard for this purpose. According to the World Health Organization^2^, a BMD value with a T-score ≤-2.5 standard deviations (SDs) in relation to the peak bone mass of young adults defines the diagnosis of osteoporosis. The T-score is applicable to postmenopausal women and men over 50 years of age and is compared to the bone density of a young, healthy population. The Z-score is used in premenopausal women and men under 50 years of age, and is compared to individuals of the same age and sex. In this population, a Z-score <-2.0 SD characterizes "low bone mass for age."

Treatment may involve non-pharmacological measures (such as dietary changes, physical activity, smoking cessation, and excessive alcohol consumption) and drug therapies. Patients at high risk of fracture, identified by tools such as Fracture Risk Assessment Tool (FRAX)^3^, or diagnosed with osteoporosis, may benefit from the use of drugs. Therapeutic options include bone antiresorptive agents (such as bisphosphonates, estrogens, and monoclonal antibodies) and anabolic agents, which stimulate bone formation.

Denosumab is a human monoclonal antibody that inhibits the binding of nuclear factor kappa B activator (receptor activator of NF-κB ligand [RANKL]), reducing bone resorption. Romosozumab, a humanized monoclonal antibody, inhibits sclerostin and promotes differentiation and osteoblastic activity, stimulating bone formation.

## OBJECTIVE

The aim of this systematic review is to evaluate the efficacy and safety of the drug romosozumab compared with denosumab when indicated for the treatment of osteoporosis in postmenopausal women and in patients with rheumatoid arthritis. Where possible, a meta-analysis of the included trials will be performed.

### Methodology

This assessment is based on scientific evidence obtained through a systematic review of the literature. When applicable, its conclusions may include a meta-analysis of common outcomes across the included studies. The description of the systematic review methodology follows the standardized checklist items of the Preferred Reporting Items for Systematic Reviews and Meta-Analyses (PRISMA) statement^4^.

### Eligibility criteria

The eligibility criteria define the specific elements required to address the clinical question outlined in the objective of this assessment. They establish the scientific rigor and consistency necessary for study inclusion, as well as the primary reasons for excluding retrieved evidence.

#### Study inclusion criteria

- Patients with osteoporosis;
- Treated with romosozumab;
- Compared with denosumab;
- Outcomes: clinically relevant efficacy and safety;
- No restrictions on study design;
- No restrictions on publication period or language;
- Abstracts with data or full-text articles.

#### Exclusion criteria

The following studies were excluded:

- Systematic reviews with or without meta-analysis and narrative revisions;
- Studies lacking extractable data on relevant outcomes (absolute numbers and/or means);
- Studies reporting only surrogate endpoints.

### Search for evidence

The searches were conducted in the following databases of published scientific information: Medline/PubMed, Cochrane Central Register of Controlled Trials (CENTRAL), and ClinicalTrials.gov (CT.gov) for unpublished registered studies.

Additional manual searches were performed in the reference lists of included studies and other relevant sources. The database search was conducted up to July 2025.

The search strategies used for each database were as follows:

- MEDLINE/PubMed-(osteoporoses OR osteoporosis OR "bone loss" OR "bone losses" OR fracture) AND (denosumab AND romosozumab);
- Cochrane CENTRAL-(romosozumab);
- ClinicalTrials.gov (CT.gov)-(osteoporoses OR osteoporosis OR bone loss OR bone losses OR fracture) AND (romosozumab).

### Assessment of risk of bias and quality of evidence

Two independent reviewers (IF and AS) will assess the risk of bias of included studies, using the Cochrane Risk of Bias Tool for non-randomized trials (RoB I)^5^ and for randomized controlled trials (RCTs) (RoB 2)^6^, rated as high, moderate, or low risk of bias.

The quality of the evidence will be assessed according to the criteria of the Grading of Recommendations Assessment, Development and Evaluation (GRADE) system^7^. The certainty of the evidence will be classified into four levels: high, moderate, low, and very low. Two reviewers (IF and AS) will assess five domains: risk of bias, inconsistency, indirect evidence, imprecision, and publication bias. In case of divergence, a third reviewer (WB) will be consulted for a final decision. The synthesis of the certainty of the evidence will be performed with the aid of the *GRADEpro Guideline Development Tool (GDT)^8^.

### Method of analysis and synthesis of the results

Data will be analyzed following the intention-to-treat principle. Categorical outcomes will be expressed as the risk difference (RD) between the intervention and control groups using the Mantel-Haenszel method. When RD is statistically significant, it will be presented with a 95% confidence interval (95%CI) and with the number needed to treat (NNT) or cause harm (NNH).

If multiple studies reported common outcomes, they were pooled through meta-analysis using the Review Manager software version 5.4 (The Nordic Cochrane Centre, The Cochrane Collaboration)^9^. The overall RD, with 95%CI, will be the final measure employed to synthesize the evidence and answer the clinical question. The estimate of the combined effect size will be performed by fixed or random effect models, according to the heterogeneity between the studies.

Statistical heterogeneity will be assessed using metric I², which quantifies the proportion of total variability attributed to heterogeneity between studies, rather than chance^10^. I² values above 50% will be considered high, and in this case, the random effects model will be applied.

### Summary of the evidence and conclusion

The evidence synthesis will be presented based on the results directly from the analyses, represented by Forest Plot graphs, considering benefits, harms, and absence of difference between the use of romosozumab and denosumab. The conclusions will primarily take into account evidence of at least moderate quality, the presence of an effect—whether beneficial or harmful—and a favorable balance between benefits and harms in patients with osteoporosis.

## RESULTS

The evidence search identified 236 studies across the Medline (PubMed), 196 in CENTRAL, and 28 in CT.gov databases. Manual searches and/or gray literature did not reveal any additional studies.

After duplicate removal and screening by title and/or abstract, 20 studies met the pre-established eligibility criteria. Of these, seven were selected for analysis of the full text. The reasons for exclusion are described in Appendix Figure 1.

Following full-text review, four studies (one randomized clinical trial and three cohort studies)^11-14^ were included to support the conclusions of this evaluation.

**Figure 1:**
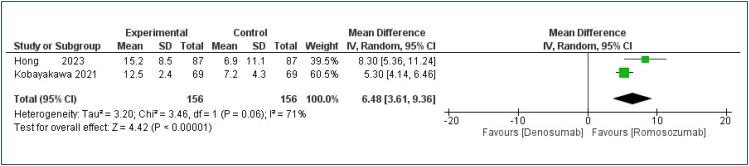
Forest plot of the comparison: romosozumab versus denosumab for percentage bone mass gain in the lumbar spine at a 12-month follow-up.

Three studies were excluded^15-17^ because they compared romosozumab with placebo or bisphosphonates, rather than with denosumab. The references of the excluded studies and the reasons for them are listed under "References." The flow diagram illustrating the selection of studies is shown in Figure 1.

The main baseline characteristics and methodological details of the included studies are described in Appendix Table 1.

### Assessment of risk of bias

Regarding the risk of bias in the pivotal study^11^ included, it was not informed whether the allocation sequence was hidden from the researchers, nor whether there was blinding of the evaluators. The study showed losses greater than 20% and did not perform a sample calculation, which resulted in a classification of high risk of bias (Appendix Table 2).

In three observational studies^12-14^ included, the propensity score matching methodology was applied to balance baseline characteristics between the groups [age, body mass index (BMI), bone mineral density (BMD), previous fractures, among others], reducing confounding bias, although not completely eliminating it—for example, non-measured variables such as physical activity level and diet. All of them had intervention bias, outcome measurement bias (due to the absence of blinding of the evaluators), and patient selection bias (they did not report the reasons for exclusions after the initial selection). The detailed results of the bias assessment of these studies are shown in Appendix Table 3.

### Results on patients with rheumatoid arthritis

Two studies^11,12^ evaluated the efficacy and safety of romosozumab compared with denosumab in patients with rheumatoid arthritis.

Mochizuki et al.^11^ conducted an RCT of 51 postmenopausal women diagnosed with osteoporosis and rheumatoid arthritis, randomizing them into two groups: 26 patients were treated with romosozumab (210 mg once monthly) and 25 with denosumab (60 mg every 6 months). After 12 months of follow-up, there was mean increase in lumbar spine BMD of 5.20% [mean difference (MD)=5.20%; 95%CI 2.73–7.67%; p<0.0001] in the romosozumab group compared to the denosumab group. There was no significant difference in femoral neck bone mass gain [MD=0.4%; 95%CI −2.21 to 3.01%; p=0.76], nor in total adverse events [RD=15%; 95%CI −4 to 34%; p=0.13].

Kobayakawa et al.^12^ used propensity score matching in a database to select patients over 18 years of age, using corticosteroids for more than three months, being treated for rheumatoid arthritis. One group of 36 patients were treated with romosozumab (210 mg/month), and another of 36 patients with denosumab (60 mg every 6 months). After 12 months, here was an increase mean of 1.2% BMD in the lumbar spine [MD=1.2%; 95%CI 0.48–1.92%; p=0.001] in romosozumab patients. However, there were no significant differences in the femoral neck [MD=0.5%; 95%CI −0.10 to 1.10%; p=0.10], fracture risk [RD=3%; 95%CI −5 to 10%] or total adverse events [RD=8%; 95%CI −2 to 18%; p=0.11].

### Results on postmenopausal osteoporosis

There were two studies^13,14^, retrospective cohorts with 312 patients, compared the efficacy and safety of romosozumab versus denosumab in women with postmenopausal osteoporosis, with a 12-month follow-up.

We found that the mean increased BMD of the lumbar spine of people taking romosozumab was 6.48% higher [MD=6.48%; 95%CI 3.36–6.46%; I^2^=71%; p<0.00001] than in people who were taking denosumab (Figure 1). However, the quality of the evidence was rated as very low (Appendix Table 4).

**Figure 2:**
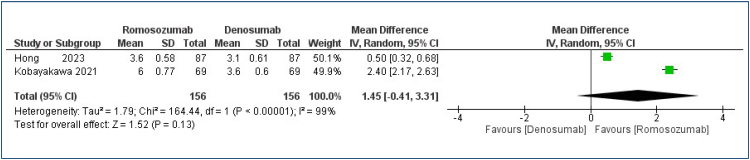
Forest plot of comparison: romosozumab versus denosumab in percentage bone mass gain in the femoral neck, for a 12-month follow-up.

There was no difference in increase of bone mass in the femoral neck between the groups compared [MD=1.45 (95%CI −0.41 to 3.31%); I^2^=99%; p<0.13] (Figure 2). Very low quality evidence (Appendix Table 4).

Kobayakawa et al.^14^, trial with 138 patients and 12-month follow-up, there was no difference in the reduction of the risk of bone fractures between the groups compared [RD=0.00; 95%CI −0.06 to 0.06; p=1.00].

Regarding the outcome of total adverse events, this trial demonstrated a significant increase in risk for patients using romosozumab, with a RD of 28% [RD=0.28; 95%CI 0.14–0.41; p<0.00001].

## SUMMARY OF THE EVIDENCE

1. Patients with osteoporosis and rheumatoid arthritis
  Comparison: romosozumab vs. denosumab (12-month follow-up)
  Randomized controlled trial:
  - Significant increase in mean BMD of the lumbar spine: 5.20% [MD=5.20% (95%CI 2.73–7.67%); p<0.0001] (quality of evidence: very low).
  - No difference in gain in femoral neck: 0.4% [MD=0.4% (95%CI −2.21 to 3.01%); p=0.76] (quality of evidence: very low).
  - No difference in total adverse events: RD=15% (95%CI −4 to 34%; p=0.13) (quality of evidence: very low).
  Observational studies:
  - Small increase in mean gain of bone mass in the lumbar spine: 1.2% [MD=1.2% (95%CI 0.48–1.92%); p=0.001] (quality of evidence: very low).
  - No difference in:
    - Femoral neck: 0.5% (95%CI −0.10 to 1.10%; p=0.10).
    - Risk of bone fracture: DR=3% (95%CI −5 to 10%; p=0.46).
    - Total adverse events: RD=8% (95%CI −2 to 18%; p=0.11).
    - (Quality of the evidence for all outcomes: very low).
2. Patients with postmenopausal osteoporosis
  Comparison: romosozumab versus denosumab (12 months follow-up)
  Observational studies:
  - Significant mean increased in bone mass in the lumbar spine: 6.48% [MD=6.48% (95%CI 3.36–6.46%); I^2^=71%; p<0.00001] (quality of evidence: very low).
  - No difference in gain in femoral neck: 1.45% [MD=1.45% (95%CI −0.41 to 3.31%); I^2^=99%; p=0.13] (quality of evidence: very low).
  - No difference in bone fracture risk reduction: RD=0.00 (95%CI −0.06 to 0.06; p=1) (quality of evidence: very low).
  - Increased risk of total adverse events by 28%: RD=0.28 (95%CI 14 to 41%; p<0.00001) (quality of evidence: very low).

## DISCUSSION

This is the first systematic review comparing the use of romosozumab and denosumab in patients with postmenopausal osteoporosis or rheumatoid arthritis.

The only included RCT^11^ demonstrated a mean increase of 5.2% BMD in the lumbar spine in elderly patients (>60 years) with rheumatoid arthritis treated with romosozumab, compared with denosumab; there was no significant increase in femoral neck BMD. However, this study did not perform sample calculations, did not blind the evaluators, and did not describe the hidden allocation method, which characterizes a high risk of bias and compromises the validity of the results.

An observational historical cohort study^12^, which assessed patients over 18 years of age using corticosteroids for the treatment of rheumatoid arthritis, reported a mean increase of 1.2% in lumbar spine BMD in the romosozumab group than in the denosumab group, also with no significant difference in the increase in femoral neck BMD. This study also showed a high risk of bias.

In patients with postmenopausal osteoporosis, the meta-analysis of two studies^13,14^ showed a mean increase of 6.48% in lumbar spine BMD in the romosozumab group than the denosumab group, with no significant difference in femoral neck.

The cohort studies^14^ showed no difference in reduction of the risk of bone fractures [RD=0.00; 95%CI −0.06 to 0.06; p=1] comparing Between the use of romosozumab to denosumab, but identified a significant 28% increase in the risk of total adverse events with the use of romosozumab [RD=0.28; 95%CI 0.14–0.41; p<0.00001]. The quality of the evidence for these outcomes was very low.

Observational studies used the propensity score matching method to minimize biases, but did not completely eliminate the influence of unmeasured variables, such as physical activity level, diet, and corticosteroid dose, which may interfere with the interpretation of the results.

In addition, all studies in this review presented the results as percentages (mean±SD), which may overestimate the effects. Since the diagnosis of osteoporosis is based on BMD SD scores (T-score ≤-2.5 SD)^2^, the conversion of the percentage gain to absolute values in SD, using the partial derivatives uncertainty propagation method^17^, can provide a more adequate estimate of the clinical impact.

For example, in the study by Kobayakawa et al.^14^, the mean gain of 5.2% in lumbar spine BMD with romosozumab (compared to denosumab) corresponds to an increase of 0.39 SD versus 0.18 SD, resulting in a difference of 0.21 SD in favor of romosozumab after 12 months. Although 5.2% seems numerically significant, the actual difference in SD (0.21) is clinically small. The relevant question is: what is the real impact of this difference on the patient's health and clinical outcomes?

### Limitations

The main limitations of this review include the small number of studies (only one RCT and three historical cohorts); the heterogeneity in the inclusion criteria and prognostic characteristics (such as age, corticosteroid dose, duration of comorbidities, and previous treatments); the presence of residual confounders, even used the propensity score matching and regression models in observational studies; Mochizuki et al.^11^ was pilot study, with a small sample size and without adequate statistical power, increasing the risk of type I error; the limitation of the interpretation of secondary outcomes (such as fractures and adverse events), which were not the primary objectives of the studies; and the short follow-up time (12 months), which is insufficient to adequately assess the long-term efficacy and safety in a chronic disease such as osteoporosis.

## CONCLUSION

The comparison between romosozumab and denosumab in patients with postmenopausal osteoporosis or rheumatoid arthritis demonstrated a small increase in lumbar spine BMD at up to 12 months, favorable to romosozumab. However, the available results present a high risk of bias and very low quality of evidence, and additional studies of greater methodological robustness are needed to confirm these findings and clarify their clinical relevance.

## Data Availability

The datasets generated and/or analyzed during the current study are available from the corresponding author upon reasonable request.
